# Benefits of combined use of ^68−^Ga Dotatoc and 5-ALA fluorescence for recurrent atypical skull-base meningioma after previous microsurgery and Gamma Knife radiosurgery: a case report

**DOI:** 10.1186/s13256-023-04023-8

**Published:** 2023-07-15

**Authors:** Iulia Peciu-Florianu, Alice Jaillard, Constantin Tuleasca, Nicolas Reyns

**Affiliations:** 1 Neurosurgery Service, CHU Lille, Roger Salengro Hospital, Lille, France; 2 Nuclear Medicine and Functional Imaging, CHU Lille, Roger Salengro Hospital, Lille, France; 3 Department of Clinical Neurosciences, Neurosurgery Service and Gamma Knife Center, Lausanne University Hospital (CHUV), Rue du Bugnon 44-46, BH-08, CH-1011 Lausanne, Switzerland; 4 Faculty of Biology and Medicine (FBM), University of Lausanne (Unil), Lausanne, Switzerland; 5 Signal Processing Laboratory (LTS 5), Ecole Polytechnique Fédérale de Lausanne (EPFL), Lausanne, Switzerland

**Keywords:** Meningioma, Atypical, Recurrent, Gamma Knife, 5-ALA, Fluorescence

## Abstract

**Background:**

Studies of novel microsurgical adjuncts, such as 5-aminolevulinic acid (5-ALA) fluorescence have shown various fluorescence patterns within meningiomas, opening new avenues for complete microsurgical resection. Here, we present a recurrent, radiation-induced meningioma, previously operated on two occasions (initial gross total resection and subtotal 12 years later) and also irradiated by Gamma Knife radiosurgery (GKR, 6 years after the first surgery). We thought to assess the usefulness of ^68−^Ga Dotatoc in surgical target planning and of 5-ALA as an adjunct for maximal microsurgical excision.

**Case report:**

We report on a 43 years-old Caucasian male diagnosed with atypical, radiation induced WHO II meningioma, with left basal temporal bone implantation. Hodgkin lymphoma treated with cranial and mediastinal radiation during infancy marked his personal history. He underwent a first gross total microsurgical resection, followed 6 and 12 years later by Gamma Knife radiosurgery (GKR) and second subtotal microsurgical resection, respectively. Magnetic resonance imaging (MRI) displayed new recurrence 13 years after initial diagnosis. He was clinically asymptomatic but routine Magnetic resonance imaging showed constant progression. There was strong ^68−^Ga Dotatoc uptake. We used 5-ALA guided microsurgical resection. Intraoperative views confirmed strong fluorescence, in concordance with both preoperative Magnetic resonance imaging enhancement and ^68−^Ga Dotatoc. The tumor was completely removed, with meningeal and bone resection.

**Conclusion:**

The authors conclude that fluorescence-guided resection using 5-ALA is useful for recurrent atypical, radiation-induced meningioma even despite previous irradiation and multiple recurrences.

## Background

Atypical meningiomas are challenging to manage due to high recurrence and poor survival rates [8]. It has been previously acknowledged that complete microsurgical resection is a major prognostic factor for better outcome [8]. In cases of incomplete excision, optimal treatment approach may include adjuvant radiation therapy in the frame of a multimodal approach [2, 8].

Fluorescence-guided surgery (FGS) using 5-aminolevulinic acid (5-ALA) has been classically used in the treatment of malignant gliomas, where the extent of resection (EOR) is a key factor for overall survival (OS) and local progression free survival (LPFS) [6]. In fact, 5-ALA FGS has become an essential adjunct in the quest for maximal resection within safe functional limits of primary infiltrating brain tumors [5]. Recently, the utility of 5-ALA has been also assessed in other neoplasms, including meningiomas, with heterogeneous results [13, 16]. Of utmost interest, a particular benefit could potentially be gained in recurrent, aggressive, atypical meningiomas, due to their invasiveness of adjacent bone, dura and vessels, further precluding complete microsurgical resection and leading to recurrences [9]. In such instances, difference between scar tissue and the tumor itself might be difficult to assess, especially after multiple treatments, including surgery and irradiation [3, 16].

The increased advances and availability of positron emission tomography/computed tomography (PET/CT) in neuro-oncology has led to the development of multiple PET radiopharmaceuticals for both diagnosis and treatment purposes. The radio-labeled amino-acid PET tracers will further bind to specific tumor-expressed receptor. Thus, they will offer an enhanced accuracy in defining the tumor-to-background contrast and further to tailor treatment approaches [19]. Recently, Gallium-68 (68 Ga) has attracted a lot of interest as an alternative positron emitter to commonly used 18 F-2-fluoro-2-deoxy-D-glucose (18 F-FDG) [14]. Meningioma cells powerfully expressing somatostatin receptor subtype 2 (SSTR 2). Such a receptor provides positron emission tomography (PET) based imaging for tumor delineation with the somatostatin-receptor ligand [^68^Ga]-DOTA-D Phe^1^-Tyr^3^-Octreotide (DOTATOC) [4]. With these properties, [68 Ga]Ga-DOTA-SSTR PET scans are commonly realized as imaging adjuncts to guide surgical resection and radiation treatment planning of complex meningiomas, refining both safe and effective target delineation [1, 15].

Here, we present a recurrent, radiation-induced meningioma, previously operated on two occasions (initial gross total resection and subtotal 12 years later) and also irradiated by Gamma Knife radiosurgery (GKR, 6 years after the first surgery). We thought to assess the usefulness of ^68−^Ga Dotatoc in surgical target planning and of 5-ALA as an adjunct for maximal microsurgical excision.

## Case report

### History and presentation

A 43 years-old Caucasian male presented with an inaugural epileptic seizure in April 2006. His neurological exam was unremarkable and seizure control was obtained with antiepileptic monotherapy.

His medical history was suggestive for Hodgkin lymphoma, treated with cranial and mediastinal radiation and chemotherapy during infancy. He was already followed for radiation-induced hypothyroidism.

**Fig. 1:**
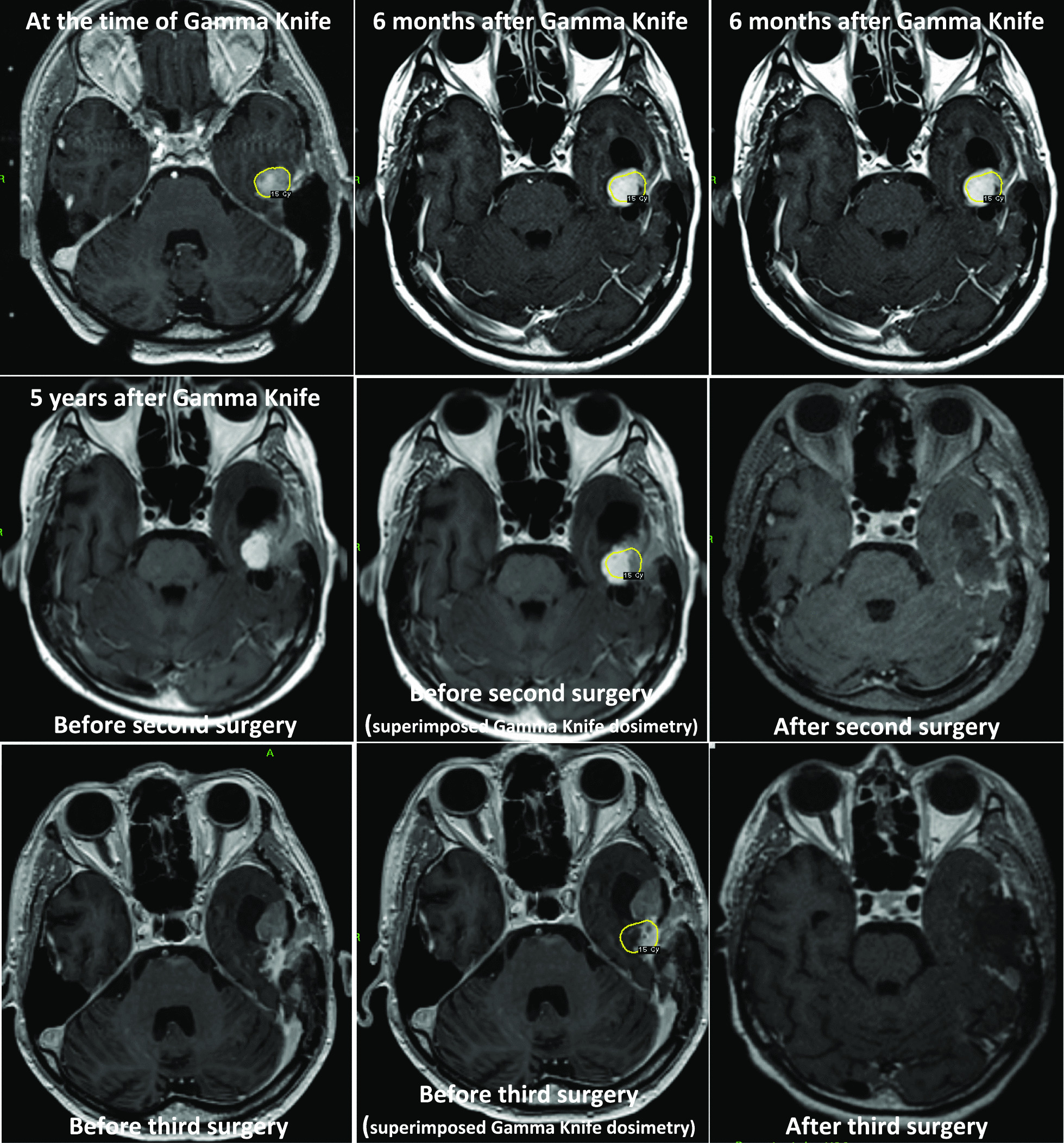
Upper part and from left to right—at the time of first GKR and 6 months later; middle part—before second surgery, without and with superimposed GKR dosimetry, colored in yellow, and after second surgery, showing the postoperative result; lower part—before third surgery, without and with superimposed GKR dosimetry, colored in yellow, and after third surgery, showing the postoperative result

**Fig. 2:**
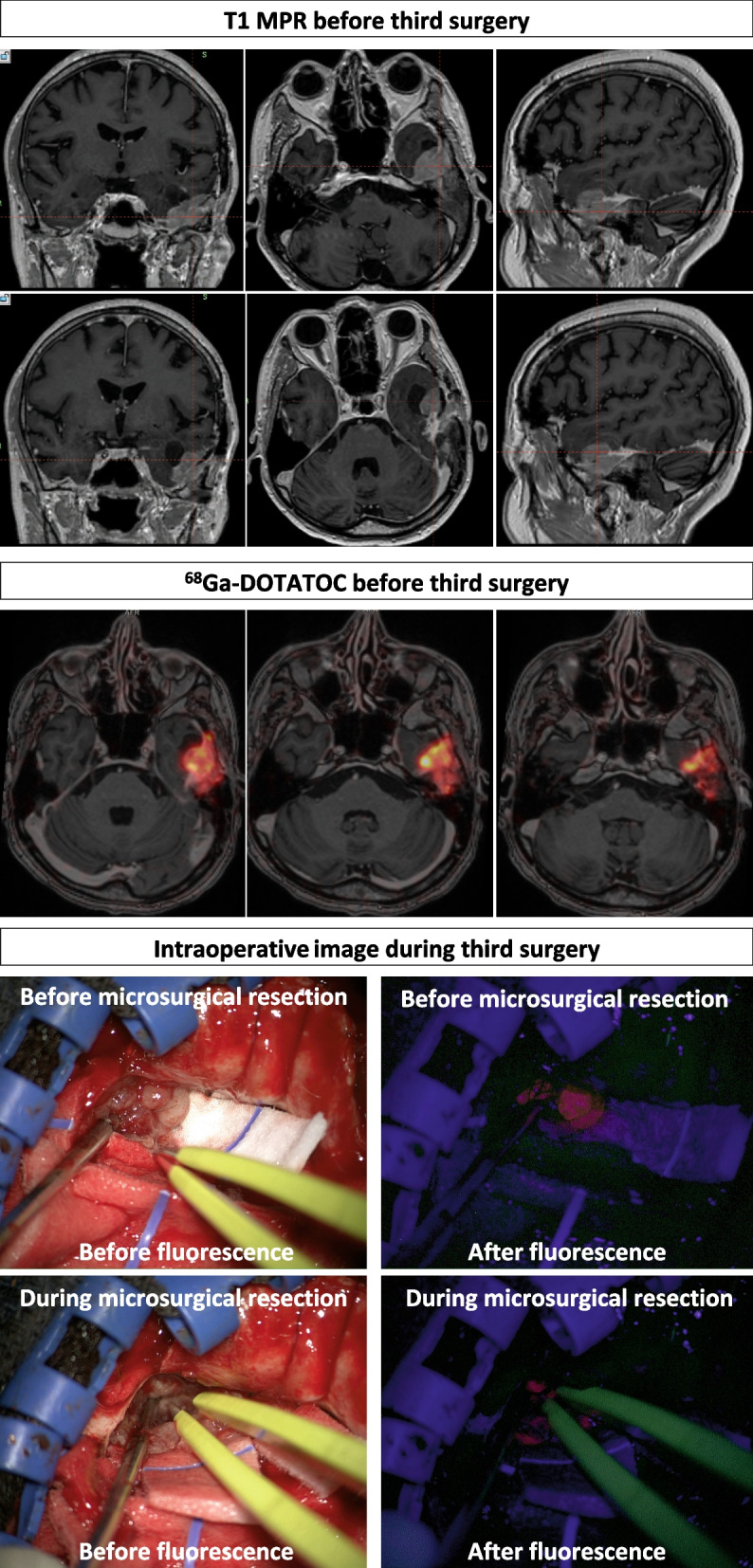
Upper part and from left to right—preoperative MRI before third surgery, in coronal, axial and sagittal plane; middle part—^68−^Ga Dotatoc before third surgery in axial plane at different levels; lower part—intraoperative images during third surgery, with and without fluorescence

Magnetic resonance imaging (MRI) showed a left skull-base temporal meningioma. The timeline of the surgical events is as followsseveral months after discovery: first gross total microsurgical resection; during the following 5 years: no further progression; six years after initial resection: major recurrence at the level of the left petrous bone. and GKR with a marginal dose of 15 Gy at the 50% isodose line (Fig. 1, upper part); up to 5 years after GKR: cyst formation in the periphery of the tumor, further stable (Fig. 1, middle part); 6 years after GKR: major nodular recurrence; 6 years after GKR: partial microsurgical resection for this recurrence (Fig. 1, middle part). Anatomopathological result confirmed a WHO grade I for the nodular progression but a WHO grade II for the previously irradiated part. Due to its personal history and previous therapeutic strategies, after multidisciplinary discussion, no additional radiation therapy was performed at this stage. Thirteen years after initial diagnosis and one year after last surgery: new recurrence (Fig. 1, lower part; Fig. 2, upper part). He was clinically asymptomatic but routine MRI showed constant progression of the nodular portion. There was strong ^68−^Ga Dotatoc enhancement, seen both at the level of tumoral nodule and dural implantation (Fig. 2, middle part). Bone infiltration was also suspected at the superior part of the petrous bone.

A basal temporal craniotomy was performed, enlarging the previous approach by drilling the temporal base and at the level of the anterior portion of the mastoid. Resection of the lesion was carried out using a fluorescence microscopy system (Carl Zeiss Pentero 900 with tumor fluorescence filter).

Four hours before the anesthesia induction, 20 mg/kg of 5-ALA was administered orally.

After dural opening the nodular segment of the meningioma was readily observed and presented bright red fluorescence (Fig. 2, lower part), allowing complete macroscopic piece-meal excision. Surrounding brain parenchyma did not present with fluorescence, permitting a sharp delineation between the meningioma and normal cerebral parenchyma. Basal dural implantation towards the petrous bone was also slightly fluorescent but no bone fluorescence was observed at this level.

Drilling was nonetheless continued in an extradural fashion, based on the neuronavigation landmarks, taking both into account the cerebral MRI performed one day preoperative and the ^68-^Ga Dotatoc results.

After complete nodular resection, the dura was excised within safe margins, based on the slight fluorescence but also on the MRI preplanning. Duraplasty was performed without any CSF leak observed at the end of surgery.

### Postoperative imaging and histological results

Postoperative MRI revealed no residual enhancement. The histopathological diagnosis confirmed WHO grade II meningioma.

### Postoperative course

Patient was discharged 5 days after surgery. His clinical exam was unremarkable.

## Discussion

We report the case of a patient with atypical, recurrent, radiation-induced, WHO grade II meningioma, multioperated and previously irradiated by GKR, who benefitted from a combined use of ^68−^Ga Dotatoc and 5-ALA fluorescence as adjuncts for a third, complete, microsurgical resection. 

Our patient initially benefited from microsurgical resection and further GKR for late recurrence. Stereotactic radiosurgery and particularly GKR is an important adjuvant in the therapeutic management of residual or recurrent atypical meningiomas, who are reputed for their invasiveness and early recurrences. Moreover, it has been also previously acknowledged that they display lower LPFS rates in response to GKR [7]. The particular location at the level of the skull base poses additional technical challenges, both in terms of microsurgical resection but also for irradiation planning [21], mainly due to vital neurovascular structures invasion and sometimes a less well-defined therapeutic target.

Meningioma recurrence after surgical resection occurs in up to 20% of cases, even if the tumor was considered histologically benign (WHO grade I) [12]. It has been previously suggested that Ga-DOTATATE uptake correlates with SSTR2 expression and offers high diagnostic accuracy to delineate meningioma from tumor-free tissue even in recurrent tumors after previous therapy [20]. In our patient’s case we also acquired a ^68−^Ga Dotatoc before the third surgery and after previous irradiation by stereotactic radiosurgery. This investigation has the advantage of high diagnostic accuracy to delineate meningioma from tumor free tissue even in recurrent tumors after previous therapy [20]. Moreover, it can be of valuable help for both the intraosseous and transosseous part [11]. In fact, our patient had a recurrence not only in the context of previous microsurgical resection but also prior GKR. Despite this, there was a strong uptake on ^68−^Ga Dotatoc [1] on preoperative neuroimaging. It is also well acknowledged that substantial recurrences are higher if subtotal removal can be achieved [10]. Thus, there is a need to improve the preoperative visualization so as to better define the exact and clear boundaries of such tumor and further attempt a gross-total/radical resection if possible, in such instances.

In the particular case of meningiomas, studies of surgical adjuncts such as 5-ALA fluorescence have suggested heterogeneous fluorescence within the meningioma, as well as nonspecific fluorescence in adjacent brain [18], thus suggesting a limited role [2]. In a recent review [18], it is questioned whether the appealing principle of achieving complete resection using 5-ALA for meningioma is really useful and it is proposed to be used only in protocolled prospective and long-term studies. However, other reports [17] suggest a higher accuracy using 5-ALA-induced fluorescence than contrast enhancement from previously obtained MRI scans, like in our present study.However, recent reports suggested its usefulness in higher-grade meningiomas [22]. Fluorescence observation allowed us to identify the extent of the nodular part of the tumor and helped avoid potentially leaving residual meningioma, especially towards the dural implantation and suspected bone invasion. Without 5-ALA guidance, we consider that it might have been difficult to perform a complete macroscopic excision. One open question remains the correlation between fluorescence heterogeneity and the mitotic index, further identifying optimal patient selection for tailored management in the near future.

## Conclusion

To the best of our knowledge, this is the first report of a recurrent, radiation-induced meningioma showing strong fluorescence in response to 5-ALA administration after previous GKR and repeated surgeries. We conclude that applying this method to such particular recurrent meningioma cases could be of valuable help for increasing the extent of resection and avoiding important neurovascular structures, especially in the skull base.

## Data Availability

Data is not available, as beside the radiological images, there is no need for further data.
